# Revisit the practice of lymph node biopsy in patients diagnosed as ductal carcinoma in situ before operation: a retrospective analysis of 682 cases and evaluation of the role of breast MRI

**DOI:** 10.1186/s12957-021-02336-w

**Published:** 2021-09-01

**Authors:** Hung-Wen Lai, Yi-Lin Chang, Shou-Tung Chen, Yu-Jun Chang, Wen-Pei Wu, Dar-Ren Chen, Shou-Jen Kuo, Chiung-Ying Liao, Hwa-Koon Wu

**Affiliations:** 1 Endoscopic & Oncoplastic Breast Surgery Center, Changhua Christian Hospital, 135 Nanxiao Street, Changhua, 500 Taiwan; 2 Division of General Surgery, Changhua Christian Hospital, 135 Nanxiao Street, Changhua, 500 Taiwan; 3 Comprehensive Breast Cancer Center, Changhua Christian Hospital, 135 Nanxiao Street, Changhua, 500 Taiwan; 4 Minimal Invasive Surgery Research Center, Changhua Christian Hospital, 135 Nanxiao Street, Changhua, 500 Taiwan; 5 Tumor Center, Changhua Christian Hospital, 135 Nanxiao Street, Changhua, 500 Taiwan; 6 Department of Surgery, Kaohsiung Medical University, Kaohsiung, Taiwan; 7 Division of Breast Surgery, Yuanlin Christian Hospital, Yuanlin, Taiwan; 8 School of Medicine, Chung Shan Medical University, Taichung, Taiwan; 9 Department of Biomedical Imaging and Radiological Sciences, Department of Surgery, School of Medicine, National Yang Ming Chiao Tung University, Taipei, Taiwan; 10 Chang Gung University College of Medicine, Taoyuan City, Taiwan; 11 Division of General Surgery, Kaohsiung Chang Gung Memorial Hospital, Kaohsiung, Taiwan; 12 Center for Research and Epidemiology, Big Data Center, Changhua Christian Hospital, 135 Nanxiao Street, Changhua, 500 Taiwan; 13 Department of Radiology, Changhua Christian Hospital, 135 Nanxiao Street, Changhua, 500 Taiwan

**Keywords:** Ductal carcinoma in situ, Upgrade, Lymph node metastasis, Sentinel lymph node biopsy (SLNB), Mastectomy

## Abstract

**Background:**

The optimal axillary lymph node (ALN) management strategy in patients diagnosed with ductal carcinoma in situ (DCIS) preoperatively remains controversial. The value of breast magnetic resonance imaging (MRI) to predict ALN metastasis pre-operative DCIS patients was evaluated.

**Methods:**

Patients with primary DCIS with or without pre-operative breast MRI evaluation and underwent breast surgery were recruited from single institution. The value of breast MRI for ALN evaluation, predictors of breast and ALN surgeries, upgrade from DCIS to invasive cancer, and ALN metastasis were analyzed.

**Results:**

A total of 682 cases with pre-operative diagnosis of DCIS were enrolled in current study. The rate of upgrade to invasive cancer were found in 34.2% of specimen, and this upgrade rate is 23% for patients who received breast conserving surgery and 40.7% for mastectomy (*p* < 0.01). Large pre-operative imaging tumor size and post-operative invasive component were risk factors to ALN metastasis. Breast MRI had 53.8% sensitivity, 77.8% specificity, 14.9% positive predictive value, 95.9% negative predictive value (NPV), and 76.2% accuracy to predict ALN metastasis in pre-OP DCIS patients. In MRI node-negative breast cancer patients with MRI tumor size < 3 cm, the NPV was 96.4%, and all these false-negative cases were N1. Pre-OP diagnosed DCIS patients with MRI tumor size < 3 cm and node negative suitable for BCS could safely omit SLNB if whole breast radiotherapy is to be performed.

**Conclusion:**

Breast MRI had high NPV to predict ALN metastasis in pre-OP DCIS patients, which is useful and could be provided as shared decision-making reference.

## Introduction

Lymph node evaluation plays important role of breast cancer staging and management, and had been evolved from axillary lymph node dissection (ALND) to sentinel lymph node biopsy (SLNB). In theory, ductal carcinoma in situ (DCIS) does not metastasize to adjacent lymph nodes, and axillary lymph node evaluation or surgery had limited role. DCIS as determined by pathologic analysis of biopsy specimens, however, does not preclude invasive disease in excised specimens, and up to 50% (range, 3.5–56%) of core needle biopsy (CNB) or vacuum-assisted core biopsy (VACB) diagnosed DCIS would upgrade to have an invasive component (IC) [1–17]. Indication and adequacy of application of SLNB in lymph node evaluation of patients with pre-operative (pre-OP) DCIS diagnosed by biopsy remained a debated issue as SLNB remains an invasive procedure and not morbidity free [18–20].

In current practice guideline, SLNB was not routinely suggested for patients with pre-OP DCIS planned to receive breast conserving surgery (BCS) as the rate of upgrade to DCIS-IC is not so high in lesion suitable for local excision, and even if invasive component found a secondary SLNB could still be performed [21]. Furthermore, according to ACOSZ0011, even 1–2 positive SLNB could be managed with whole breast radiotherapy in T1–T2 tumor received BCS without ALND [22]. In patients with pre-OP DCIS and indicated for mastectomy, however, SLNB remained recommended as the upgrade rate is increased and there would be rare chance for secondary lymph node surgical biopsy. In recent studies, the rate of positive SLNB following mastectomy of patients with pre-OP biopsied DCIS was low (around 10–20%), which raising the question of the need and value of routine SLNB in this particular group of patients in modern era of breast imaging [1, 23–28].

In our previous study, we had showed pre-OP breast magnetic resonance image (MRI) had clinical benefit in predict DCIS with invasive component (DCIS-IC) [29–33]. Certain characteristics of breast MRI, like MRI evidence of nipple areolar complex invasion, mass-like lesions, and measurable apparent diffusion coefficient area were significant predictors of DCIS-IC [34]. DCIS-IC was found to be the most important predictor of ALN metastasis in SLNB in patients with pre-OP DCIS. We hypothesized that pre-OP MRI could have potential role for ALN evaluation and prevent unnecessary SLNB in some conditions [35].

The aim of current study is to evaluate the role of ALN surgery in pre-OP biopsy diagnosed DCIS patients, and investigate the accuracy of breast MRI to predict ALN metastasis. The rate and predictors of upgrade from pre-OP DCIS to DCIS-IC, ALN metastasis, and potential of breast MRI to replace SLNB in pre-OP biopsy diagnosed DCIS patients would be analyzed and discussed.

## Materials and methods

### Patients

**Fig. 1:**
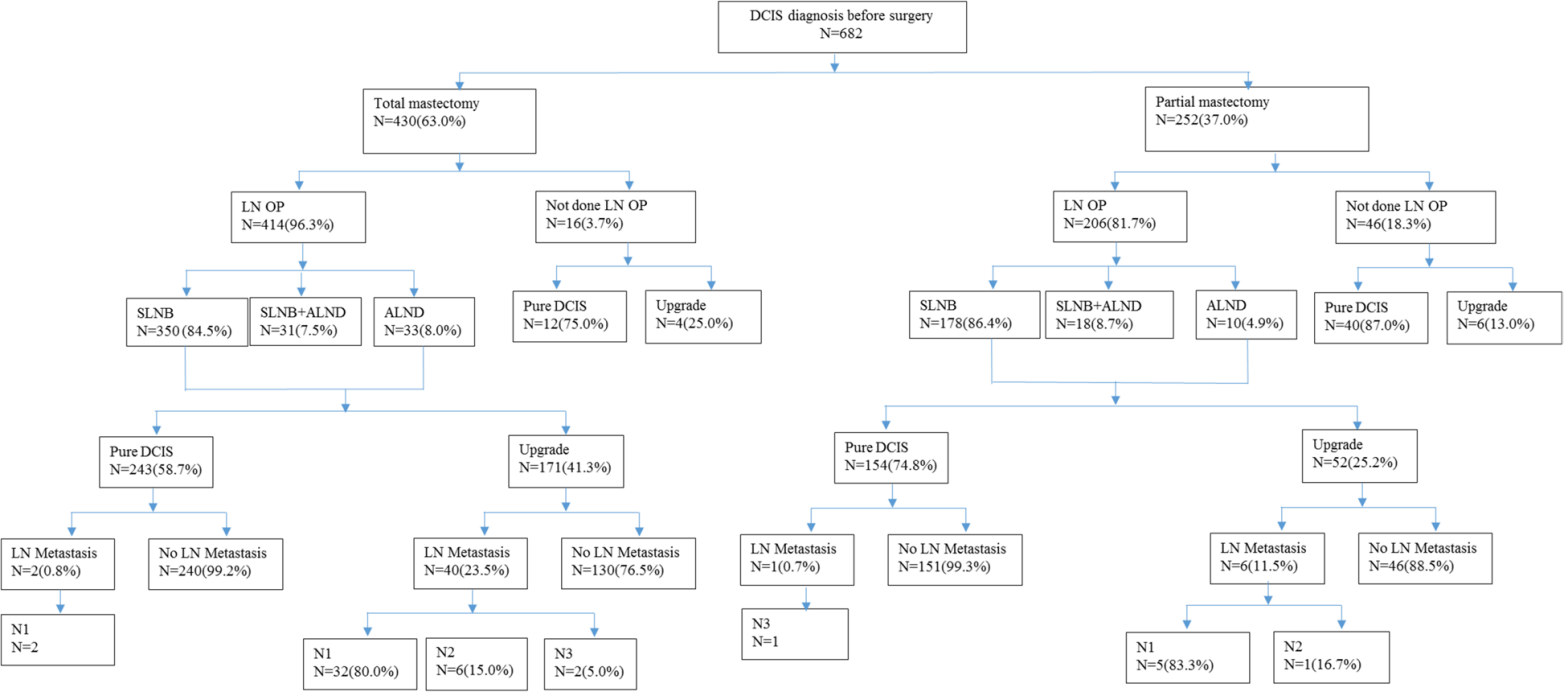
Flow chart of patients management of 682 pre-operative biopsy diagnosed ductal carcinoma in situ cases. The number of patients allocation to either breast conserving surgery (BCS) or mastectomy, whether received axillary lymph node surgery, pathology upgrade to invasive component or not, and post-operative lymph node status were described in details

Patients with primary DCIS as diagnosed by biopsy (mainly CNB, VACB, or excision biopsy) with or without pre-OP breast MRI evaluation and underwent breast surgery during the period of January 2009 to December 2018 at the Changhua Christian Hospital (CCH), a tertiary medical center in central Taiwan, were retrospectively recruited from the breast cancer database. Patients without detailed clinicopathologic data were excluded (Fig. 1). The study was approved by the institutional review board (IRB) of CCH (CCH IRB No. 140404 and No. 210519). The clinicopathologic factors gathered from the data base included age, tumor size, biopsy method, tumor grade, and status of estrogen receptor (ER), progesterone receptor (PR), and human epithelial growth factor receptor 2 (HER-2) expression.

### Defining the adequacy of sentinel lymph node biopsy (SLNB)

In patients whom SLNB was indicated, methylene blue and/or radioisotope (Tc99m) were used. Indication and threshold for surgical lymph biopsy in patients with pre-OP DCIS patients remained a controversial issue. When IC present, SLNB could be viewed as “adequate-treatment” while no axillary surgery seemed likely to “under-treatment”. When pure DCIS found in permanent pathology, SLNB seemed “over-treatment” and patients without surgical lymph node biopsy viewed as “adequate-treatment”. We apply the above mentioned: “adequate-treatment, under-treatment, over-treatment” to our patients according to their post-operative pathology and whether ALN surgery was performed.

To evaluate the indication and adequacy of surgical lymph node biopsy of patients with pre-OP biopsy diagnosed DCIS patients, another group of pre-OP invasive cancer patients were identified for comparison. The risk of final ALN metastasis, the degree of ALN metastasis (N1 (1–3 nodes), N2(4–9 nodes), and N3(> 10 nodes)) were compared between different groups of patients, namely pre-OP DCIS, post-OP pure DCIS, DCIS-IC, and invasive cancer patients.

### Predictors for upgrade from DCIS to DCIS with invasive component (DCIS-IC)

Patients were separated into two groups, namely a DCIS group comprising patients with post-operative (post-OP) histopathologic evidence of pure DCIS and a DCIS-IC group comprising patients with post-OP evidence of DCIS with invasive component (i.e., basement membrane invasion, characterized immunohistochemically by the lack of p63 staining in myoepithelial cells).

### Magnetic resonance imaging (MRI) and protocol

The sensitivity, specificity, positive predictive value (PPV), negative predictive value (NPV), and accuracy of breast MRI in prediction of ALN metastasis was evaluated by comparing the concordance of pre-operative breast MRI lymph node report and post-operative pathologic lymph node status. The protocol and method of breast MRI used in current study had been reported before [34], and a brief summary was described. MR imaging was performed with a Siemens MAGNETOM Verio3.0 Tesla MRI machine. All patients were imaged in the prone position with both breasts placed into a dedicated 16-channel breast coil.

MR imaging protocols included the following: bilateral laxial turbo-spin-echo fat-suppressed T2-weighted imaging (TR/TE4630/70 ms; field of view 320 mm; slice thickness 3 mm; number of excitations 1), axial turbo-spin-echo T1-weighted imaging (TR/TE736/9.1 ms; field of view 320 mm; slice thickness 3 mm; number of excitations 1), and diffusion-weighted imaging (DWI) (TR/TE5800/82 ms; field of view 360 mm; slice thickness 3 mm, with *b* values of 0, 400, and 800 s/mm^2^). Dynamic contrast-enhanced MR images (DCE-MRI) were obtained with a three-dimensional fat-suppressed volumetric interpolated breath-hold examination (VIBE) sequence with parallel acquisition once before and five times after a bolus injection of gadobenate dimeglumine (0.1 mmol/kg). Both breasts were examined in the transverse plane at 60 s intervals in each phase of the dynamic studies. All of the MRI readings were interpreted by an experienced, board-certified breast imaging radiologist (HKW), who had a 35-year radiologist career and 15 years of experience of breast MRI.

### Statistical analyses

Data are expressed as mean ± standard deviation for continuous variables. Categorical variables were compared using the chi-square test or Fisher’s exact test when appropriate. The independent *t* test was used to compare continuous variables. A *P* value of less than 0.05 was considered to indicate statistical significance; all tests were two-tailed. All statistical analyses were performed on a personal computer with the statistical package SPSS for Windows (Version19.0, SPSS, Chicago).

## Results

According to inclusion and exclusion criteria, a total of 682 cases with pre-OP biopsy diagnosed DCIS were enrolled in current study. Among our patients, 252 (37%) received BCS while 430 (63%) received mastectomy, and surgical lymph node biopsy was performed in 90.9% of them. The clinicopathologic characteristics and management flow chart of pre-OP DCIS patients were shown in Table 1 and Fig. 1. Post-OP upgrade to invasive breast cancer were found in 34.2% (233/682) breast cancer specimens, and this upgrade rate is 23% (58/252) for patients received BCS and 40.7% (175/430) mastectomy (Table 2, *p* < 0.01).

**Table 1:** Clinical presentations and demographic data of 682 patients with pre-operative diagnosis of ductal carcinoma in situ (DCIS)

***N* = 682**	Mean ± SD (%)
Age, year	52.3 ± 10.1
Location	
Right	328(48.1)
Left	354(51.9)
Biopsy method(N/A = 3)	
CNB	421(62)
Stereotactic biopsy	167(24.6)
Excisional biopsy	88(12.9)
MMG	3(0.4)
Tumor size, cm	2.2 ± 2.5
Lymph node(N/A = 33)	
Positive	49(7.6)
Negative	600(92.4)
Stage (N/A = 33)	
0	390(60.1)
I	183(28.2)
II	65(10.0)
III	11(1.7)
Lymph node stage(N/A = 83)	
N0	549(91.7)
N1	40(6.7)
N2	7(1.2)
N3	3(0.5)
Grade(N/A = 74)	
I	76(12.5)
II	337(55.4)
III	195(32.1)
ER(N/A = 24)	
Positive	484(73.6)
Negative	174(26.4)
PR(N/A = 33)	
Positive	438(67.5)
Negative	211(32.5)
HER-2(N/A = 203)	
Positive	178(37.2)
Negative	301(62.8)
Post-OP pathology	
DCIS	412(60.4)
LCIS	5(0.7)
DCIS + LCIS	7(1.0)
DCIS + microinvasive	5(0.7)
DCIS + tubular carcinoma	1(0.1)
DCIS + mucinous carcinoma	3(0.4)
IDC + DCIS	228(33.4)
IDC	9(1.3)
ILC	1(0.1)
Other	11(1.6)

*NA* not available, *CNB* core needle biopsy, *MMG* mammography guided biopsy, *ER* estrogen receptor, *PR* progesterone receptor, *HER-2* human epithelial receptor type 2, *OP* operation, *DCIS* ductal carcinoma in situ, *LCIS* lobular carcinoma in situ, *ILC* infiltrating lobular carcinoma, *IDC* infiltrating ductal carcinoma

**Table 2:** Upgrade rate, adequacy of surgical lymph node biopsy, and rate of lymph node metastasis in patients diagnosed as ductal carcinoma in situ before operation

Upgrade ratio versus operation method					
	BCS	Mastectomy	Total	P value	
DCIS	194	255	449(65.8%)	<0.01	
DCIS-IC	58	175	233(34.2%)		
Total	252 (37%)	430 (63%)	682		
Post-OP pathology and lymph node surgery					
Post-OP pathology	DCIS	DCIS-IC	Total	P value	
Axillary surgery					
ALN biopsy/dissection	397(58.2%) over treatment	223(32.7%) adequate treatment	620	0.01	
No axillary surgery	52(7.6%) adequate treatment	10(1.5%) under-treatment	62		
Total	449	233	682(100%)		
Pathology and lymph node metastasis distribution					
	Pre-OP DCIS N=682	Pure-DCIS N=449	DCIS-IC N=233	Pre-OP invasive cancer N=2268	P value
No LN metastasis	92.4% (600/649)	99.2% (394/397)	79.4% (177/223)	61% (1385/2268)	<0.01
LN metastasis	7.6% (49/649)	0.8% (3/397)	20.6% (46/223)	39% (883/2268)	
No axilla surgery	N=33	N=52	N=10	N=0	
N1	0	1 ITC, 1micro-metastasis	80.4% (37/46)	68% (600/883)	
N2	0	0	15.2% (7/46)	21.9% (193/883)	
N3	0	1	4.3% (2/46)	10.2% (90/883)	
Breast MRI lymph node metastasis evaluation					
	MRI predict LN metastasis		MRI predict LN negative		Total
Pathology LN metastasis	14		12		26
Pathology LN negative	80		280		360
Total	94		292		386
Sensitivity=14/26=53.8%					
Specificity=280/360=77.8%					
Positive predictive value (PPV)=14/94=14.9%					
Negative predictive value (NPV)=280/292=95.9%					
Accuracy= (14+280)/386 =76.2%					

*DCIS* ductal carcinoma in situ, *DCIS-IC* ductal carcinoma in situ with invasive component, *BCS* breast conserving surgery, *ALN* axillary lymph node, *OP* operation, *LN* lymph node, *ITC* isolated tumor cell

The risk of ALN metastasis rate is overall 7.6% in pre-operative DCIS patients, 0.8% in post-operative pure DCIS, and 20.6% in post-operative DCIS-IC. The rate and distribution of ALN metastases of 682 patients with pre-OP diagnosed DCIS were compared with another cohort of 2268 pre-OP diagnosed invasive cancer patients and summarized in Table 2. According to post-OP pathology and whether SLNB was performed, 32.6% of SLNB was rated as “adequate-treatment”, 58.4% “over-treatment”, and 1.5% patients “under-treatment” while 7.6% patients with post-OP pure DCIS did not receive SLNB (“adequate-management”, Fig. 1 and Table 2).

About 386 with detail pre-OP MRI ALN evaluation and post-OP pathologic report were analyzed for concordance, and MRI had 53.8% sensitivity, 77.8% specificity, 14.9% PPV, 95.9% NPV, and 76.2% overall accuracy (Table 2). In pre-OP MRI evaluated tumor size < 3 cm and no sign of ALN node metastasis breast cancer patients, the NPV was 96.4%, and all the 3.6% (5/137) false-negative (FN) cases were N1 patients. Among 70 patients that received BCS, only 1 patient was found to have lymph node metastasis (N1:1/22). Another 67 patients received mastectomy, and 4 patients were found to have lymph node metastasis (all N1: 2/13, 1/5, 1/2, 1/3).

The differences between patients who received breast (BCS versus mastectomy) and lymph node surgeries were compared and shown in Table 3. Larger pre-OP imaging tumor size is an independent risk factor for mastectomy and surgical ALN biopsy. The rate of surgical ALN biopsy rate is 96.3% (414/430) in mastectomy group, and 81.7% (206/252) in BCS (partial mastectomy) group (*P* < 0.01).OP method was also related to upgrade to DCIS, patients received total mastectomy had higher upgrade rate than partial mastectomy (BCS) patients (40.7% versus 23%, *P* < 0.01, Table 3).

**Table 3:** Predictors of patients’ selection for breast conserving surgery, mastectomy, and axillary lymph node surgery

	Total mastectomy versus partial mastectomy (BCS)			Lymph node surgery versus no surgery		
Pre-OP DCIS (*N* = 682)	Total mastectomy *N* = 430	Partial mastectomy *N* = 252	*P* value	No LN surgery *N* = 62	With LN surgery *N* = 620	*P* value
Age, year	51.9 ± 9.9	53.1 ± 10.5	0.14	54 ± 12.2	52.2 ± 9.9	0.17
Location			0.33			0.31
Right	213(49.5)	115(45.6)		26(41.9)	302(48.7)	
Left	217(50.5)	137(54.4)		36(58.1)	318(51.3)	
Biopsy method	N/A = 3	N/A = 0	< 0.01	N/A = 0	N/A = 3	< 0.01
CNB	298(69.8)	123(48.8)		19(30.6)	402(65.2)	
Stereotactic biopsy	84(19.7)	83(32.9)		23(37.1)	144(23.3)	
Excisional biopsy	43(10.1)	45(17.9)		18(29)	70(11.3)	
MMG	2(0.5)	1(0.4)		2(3.2)	1(0.2)	
Sonogram tumor size, cm	2.2 ± 1.2	1.6 ± 0.8	< 0.01	1.7 ± 1.4	2 ± 1.1	0.11
Mammogram tumor size, cm	2.9 ± 1.3	2.0 ± 0.9	< 0.01	1.6 ± 0.4	2.6 ± 1.2	0.10
MRI tumor size, cm	4.5 ± 2.3	2.8 ± 1.4	< 0.01	2.7 ± 1.7	4 ± 2.2	< 0.01
Pathology tumor size^a^, cm (cm)	2.6 ± 2.9	1.7 ± 1.6	< 0.01	1.8 ± 1.7	2.3 ± 2.6	0.22
Gross tumor size, cm^b^ (invasive + non-invasive)	4 ± 2.6	2.2 ± 1.4	< 0.01	2.6 ± 1.8	3.5 ± 2.4	0.04
Stage	N/A = 11	N/A = 22	< 0.01	N/A = 12	N/A = 21	0.03
0	226(53.9)	164(71.3)		40(80)	350(58.4)	
I	130(31)	53(23)		8(16)	175(29.2)	
II	54(12.9)	11(4.8)		2(4)	63(10.5)	
III	9(2.1)	2(0.9)		0(0)	11(1.8)	
Grade	N/A = 38	N/A = 36	< 0.01	N/A = 19	N/A = 55	0.14
I	39(9.9)	37(17.1)		6(14.0)	70(12.4)	
II	215(54.8)	122(56.5)		29(67.4)	308(54.5)	
III	138(35.2)	57(26.4)		8(18.6)	187(33.1)	
ER	N/A = 9	N/A = 15	< 0.01	N/A = 11	N/A = 13	0.25
Positive	293(69.6)	191(80.6)		41(80.4)	443(73.0)	
Negative	128(30.4)	46(19.4)		10(19.6)	164(27.0)	
PR	N/A = 13	N/A = 20	< 0.01	N/A = 11	N/A = 22	0.15
Positive	259(62.1)	179(77.2)		39(76.5)	399(66.7)	
Negative	158(37.9)	53(22.8)		12(23.5)	199(33.3)	
HER-2(N/A = 203)	N/A = 121	N/A = 82	0.53	N/A = 29	N/A = 174	0.64
Positive	118(38.2)	60(35.3)		11(33.3)	167(37.4)	
Negative	191(61.8)	110(64.7)		22(66.7)	279(62.6)	
Subtype	N/A = 68	N/A = 52	< 0.01	N/A = 24	N/A = 96	0.17
Luminal A	198(54.7)	133(66.5)		27(71.1)	304(58.0)	
Luminal B1	23(6.4)	11(5.5)		2(5.3)	32(6.1)	
Luminal B2	50(13.8)	32(16.0)		7(18.4)	75(14.3)	
HER-2( +)	56(15.5)	15(7.5)		2(5.3)	69(13.2)	
TNBC	35(9.7)	9(4.5)		0(0)	44(8.4)	
Post-OP pathology			< 0.01			< 0.01
DCIS	255(59.3)	194(77)		52(83.9)	397(64)	
Upgrade	175(40.7)	58(23)		10(16.1)	223(36)	
Ki-67	N/A = 313	N/A = 221	< 0.01	N/A = 57	N/A = 477	0.92
≦ 20	67(57.3)	25(80.6)		3(60)	89(62.2)	
> 20	50(42.7)	6(19.4))		2(40)	54(37.8)	
MRI LN metastasis	NA = 166	N/A = 109	< 0.05	NA = 28	N/A = 247	0.10
Yes	69(26.1)	25(17.5)		30(88.2)	90(24.1)	
No	195(73.9)	118(82.5)		4(11.8)	283(75.9)	
Lymph node stage	N/A = 60	N/A = 23	< 0.01	N/A = 15	N/A = 68	0.20
N0	327(88.4)	222(96.9)		47(100)	502(90.9)	
N1	35(9.5)	5(2.2))		0(0)	40(7.2)	
N2	6(1.6)	1(0.4)		0(0)	7(1.3)	
N3	2(0.5)	1(0.4)		0(0)	3(0.5)	
LN metastasis	N/A = 8	N/A = 25	< 0.01	N/A = 29	N/A = 4	0.09
Yes	42(10)	7(3.1)		0(0)	49(8)	
No	380(90)	220(96.9)		33(100)	567(92)	
OP method	–	–	–			< 0.01
Total	–	–	–	16(25.8)	414(66.8)	
Partial	–	–	–	46(74.2)	206(33.2)	
Method LN			< 0.01	–	–	–
SLNB	350(81.4)	178(70.6)		–	–	–
SLNB + ALND	31(7.2)	18(7.1)		–	–	–
ALND	33(7.7)	10(4)		–	–	–
ND	16(3.7)	46(18.3)		–	–	–

*MMG* mammography guided biopsy, *LN* lymph node, *SLNB* sentinel lymph node biopsy, *ALND* axillary lymph node dissection, *ND* not done, *OP* operation, *DCIS* ductal carcinoma in situ, *TNBC* triple negative breast cancer, *ER* estrogen receptor, *PR* progesterone receptor, *HER-2* human epithelial grow receptor type 2, *N/A* not available

^a^Pathology tumor size: in pure DCIS cases pathologic tumor size=DCIS tumor size, if containing invasive component then pathologic tumor size=invasive cancer tumor size

^b^Gross tumor size: invasive + non-invasive component tumor size

Predictors for post-OP upgraded to DCIS-IC were shown in Table 4, and larger tumor size, ER, and/or PR-negative tumors were associated with upgrade to DCIS-IC. Predictors for LN metastasis for patients with pre-OP diagnosed DCIS were evaluated, pre-OP imaging tumor size, DCIS-IC, and MRI predicted lymph node metastasis were risk factors to lymph node metastasis. In multivariate analysis, imaging tumor size (odds ratio, OR = 1.93), DCIS-IC (OR = 34.9) remained important factors (Table 5).

**Table 4:** Predictors for upgrade to ductal carcinoma in situ (DCIS) with invasive component (IC) in pre-operative biopsy diagnosed DCIS patients

*N* = 682	Post-OP pure DCIS (*N* = 449)	Post-OP upgrade to DCIS-IC (*N* = 233)	*P* value
Age, year	52 ± 9.8	52.9 ± 10.8	0.29
Location			0.62
Right	219(48.8)	109(46.8)	
Left	230(51.2)	124(53.2)	
Biopsy method	N/A = 2	N/A = 1	< 0.01
CNB	233(52.1)	188(81)	
Stereotactic biopsy	140(31.3)	27(11.6)	
Excisional biopsy	71(15.9)	17(7.3)	
MMG	3(0.7)	0(0)	
Sonogram tumor size, cm	1.9 ± 1.1	2.2 ± 1.2	< 0.01
Mammogram tumor size, cm	2.4 ± 1.3	2.8 ± 1.1	0.14
MRI tumor size, cm	3.6 ± 2.2	4.5 ± 2.1	< 0.01
Pathology tumor size^a^, cm	2.7 ± 2.7	1.4 ± 1.9	< 0.01
Gross tumor size^b^, cm	3.3 ± 2.4	3.8 ± 2.4	0.02
Stage (N/A = 33)	N/A = 31	N/A = 2	< 0.01
0	381(91.1)	9(3.9)	
I	26(6.2)	157(68.0)	
II	10(2.4)	55(23.8)	
III	1(0.2)	10(4.3)	
Grade (N/A = 74)	N/A = 44	N/A = 30	< 0.01
I	28(6.9)	48(23.6)	
II	211(52.1)	126(62.1)	
III	166(41.0)	29(14.3)	
ER	N/A = 24		< 0.01
Positive	335(78.8)	149(63.9)	
Negative	90(21.2)	84(36.1)	
PR (N/A = 33)	N/A = 33		< 0.01
Positive	303(72.8)	135(57.9)	
Negative	113(27.2)	98(42.1)	
HER-2 (N/A = 203)	N/A = 199	N/A = 4	0.25
Positive	99(39.6)	79(34.5)	
Negative	151(60.4)	150(65.5)	
MRI LN metastasis	N/A = 184	N/A = 91	0.01
Yes	50(18.9)	44(31.0)	
No	215(81.1)	98(69.0)	
Lymph node stage	N/A = 61	N/A = 22	< 0.01
N0	385(99.2)	164(77.7)	
N1	2(0.5)	38(18.0)	
N2	0(0)	7(3.3)	
N3	1(0.3)	2(0.9)	
OP method			< 0.01
Total	255(56.8)	175(75.1)	
Partial	194(43.2)	58(24.9)	
LN meta	N/A = 30	N/A = 3	< 0.01
Yes	3(0.7)	46(20.0)	
No	416(99.3)	184(80.0)	
Method_LN			< 0.01
SLNB	365(81.3)	163(70.0)	
SLNB + ALND	14(3.1)	35(15.0)	
ALND	18(4.0)	25(10.7)	
ND	52(11.6)	10(4.3)	

*MMG* mammography-guided biopsy, *LN* lymph node, *SLNB* sentinel lymph node biopsy, *ALND* axillary lymph node dissection, *ND* not done, *OP* operation, *DCIS* ductal carcinoma in situ, *TNBC* triple negative breast cancer, *ER* estrogen receptor, *PR* progesterone receptor, *HER-2* human epithelial grow receptor type 2, *N/A* not available

^a^Pathology tumor size: in pure DCIS cases pathologic tumor size=DCIS tumor size, if containing invasive component then pathologic tumor size=invasive cancer tumor size,

^b^Gross tumor size: invasive + non-invasive component tumor size

**Table 5:** Univariate and multivariate for factors related to lymph node metastasis in patients with pre-operative biopsy diagnosed ductal carcinoma in situ

	**Univariate analysis**			**Multivariate analysis**		
	Odds ratio	95% CI	*P*	Odds ratio	95% CI	*P*
Age	0.97	0.94–1	0.06			
Pathology^a^ tumor size (cm) overall group	1.01	0.9–1.13	0.9			
Pathology^a^ DCIS-IC invasive part tumor size (invasive, cm) excluding pure DCIS cases	1.32	1.1–1.59	< 0.01			
MRI tumor size (cm)	1.21	1–1.42	0.02			
Sonogram tumor size (cm)	1.8	1.4–2.3	< 0.01	1.93	1.23–3.03	< 0.01
Mammogram tumor size (cm)	1.78	1–3.09	0.04			
Pathology gloss^b^ tumor size ( cm)	1.03	0.91–1.16	0.63			
Pathology-DCIS-IC gloss^b ^tumor size ( cm)	0.99	0.86–1.13	0.86			
Pathology-pure DCIS gloss^b^ tumor size ( cm)	0.54	0.15–1.99	0.36			
Post OP biopsy (IDC) VS DCIS	34.94	10.73–113.79	< 0.01	36.37	4.63–288.81	< 0.01
Grade (II, III)VS I	1.1	0.42–2.88	0.85			
ER (positive) VS negative	0.61	0.33–1.11	0.1			
PR (positive) VS negative	0.7	0.38–1.26	0.23			
HER-2 (positive) VS negative	0.89	0.47–1.68	0.72			
Ki -67 (> 20)VS ≦ 20	1.64	0.71–3.81	0.25			
MRI lymph node metastasis (Y)VS N	4.08	1.82–9.18	< 0.01	2.2	0.83–5.83	0.11

*MMG* mammography guided biopsy, *LN* lymph node, *SLNB* sentinel lymph node biopsy, *ALND* axillary lymph node dissection, *ND* not done, *OP* operation, *DCIS* ductal carcinoma in situ, *TNBC* triple negative breast cancer, *ER* estrogen receptor, *PR* progesterone receptor, *HER-2* human epithelial grow receptor type 2, *N/A* not available

^a^Pathology tumor size: in pure DCIS cases pathologic tumor size=DCIS tumor size, if containing invasive component then pathologic tumor size=invasive cancer tumor size

^b^Gross tumor size: invasive + non-invasive component tumor size

**Fig. 2:**
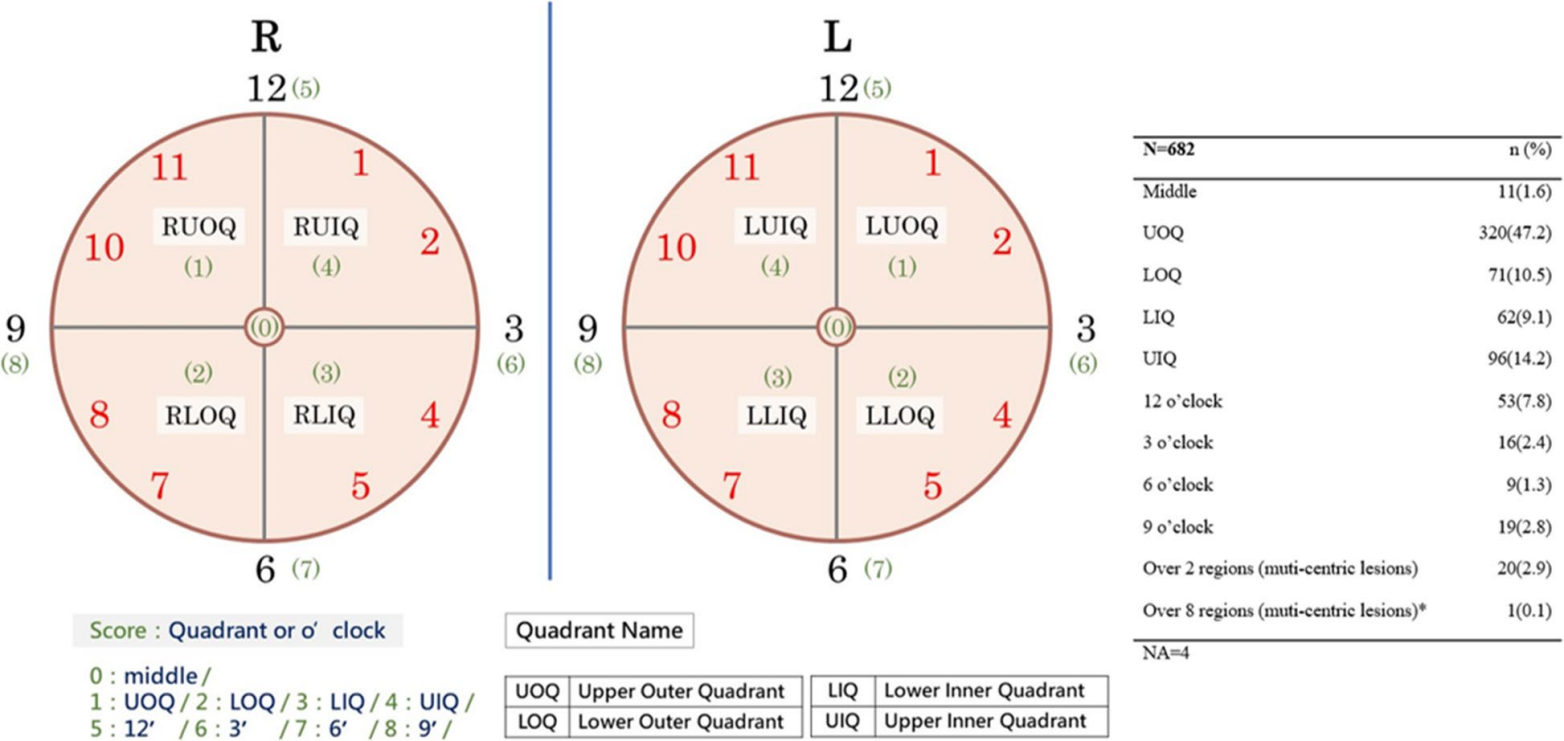
The score of the tumor location for four quadrant and 12 o’clock

The locations of breast cancer for patients diagnosed with pre-OP DCIS were listed and categorized in Fig. 2. Almost half of pre-OP DCIS patients of location were in the upper outer quadrant of the breast, and only 3% of these patients were multi-centric lesion.

## Discussion

In the current study, we enrolled 682 pre-OP DCIS patients and compared ALN metastasis pattern with another cohort of 2268 pre-OP diagnosed invasive cancer. We found 34.2% of these pre-OP DCIS patients upgraded to DCIS-IC in final pathology, and this upgrade rate was consistent with literate reported range (3.5–56%)[1–17]. The risk of ALN metastasis rate varied widely depends on the pre-operative pathology, and in our current study, the ALN metastasis rate is 7.6% in pre-OP DCIS patients, and up to 39% in pre-OP invasive cancer group (Table 2). In patients with post-OP pure DCIS, the LN metastasis rate is lowest (0.8%), and ALN metastasis was found in 20.6% of patients with DCIS-IC. We also demonstrated that the ALN disease burden (N1-3) varied among different categories of patients (Table 2). These data reminded us the differences of ALN metastatic risk in each category of patients, and more tailored or individualized ALN evaluation strategy should be offered.

Indication and threshold for surgical lymph biopsy in patients with pre-OP biopsy diagnosed DCIS remained a controversy issue for decades. When invasive component is present, SLNB was viewed as “adequate-treatment” while no axillary surgery seemed likely to “under-treatment”. When pure DCIS found in permanent pathology, SLNB seemed “over-treatment”. Under this concept, in our pre-OP DCIS patients, 32.6% were adequate-treated with SLNB, 58.4% over-treated, and 1.5% under-treated. Another 7.6% patients with post-OP pure DCIS did not receive surgical axillary biopsy, which should view as adequate-observation. If only “invasive component” present in final pathology viewed as an indication for SLNB, then up to 60% of patients diagnosed with pre-OP DCIS received inadequate axillary management (Table 2). This highlights the “unmet medical need” in modern era of personalized breast cancer care, which over-treated the low risk and/or low disease burden axilla.

In post-OP pure DICS patients, 0.8% ALN metastasis rate is not justified for surgical ALN biopsy. In pre-OP invasive cancer patients, ALN metastasis rate could up to 39% and surgical lymph node biopsy with either SLN and/or ALND is indicated (Table 2). The group of patients, who diagnosed initially as DCIS, had a substantial risk of upgrade to invasive cancer (34.2% in current study) and 7.6% overall lymph node metastasis cases detected. In patients of DCIS-IC, the ALN metastasis rate up to 20.6%. One could speculate that patients with biopsy proven pre-OP DCIS present a category of “low” (pure DCIS) to “intermediate” (DCIS-IC) risk of ALN metastasis, which possessed some controversy whether routine lymph node surgery should be performed and it remained unsolved issue even in current modern breast imaging era.

According to NCCN and other practice guideline, patients with pre-OP biopsy diagnosed DCIS, selected for BCS and adjuvant radiotherapy, do not routinely indicate for SLNB [36]. As most patients (77% in current study) with pre-OP biopsy diagnosed DCIS received BCS would remain pure DCIS in final pathology report (Fig. 1, Table 2). Furthermore, according to ACOSZ0011 trial, even 1–2 positive sentinel lymph node present, patients could omit ALND if breast cancer with tumor size T1–T2, received BCS and whole breast radiotherapy would be performed [22]. These reasons supported that SLNB is not routinely needed in pre-OP biopsy diagnosed DCIS patients indicated for BCS (Table 2).

However, some patients with pre-OP biopsy diagnosed DCIS and indicated for BCS would have post-op upgrade to DCIS-IC and with more than 2 lymph nodes metastases. In current study, we observed 7 pre-OP DCIS patients who received BCS presented with ALN metastases in final pathology check-up. Five of them were N1, 1 was N2, and the other 1 was N3 status (Fig. 1). This accounts that about 2.7% (7/252) of pre-OP DCIS and indicated BCS patients would have ALN metastasis. Although only few patients would have > 2 occult metastatic lymph nodes, routinely abandon SLNB is also worrisome.

Controversy did persist about the role of SLNB in patients with pre-OP biopsy diagnosed DCIS and indicated for mastectomy. Parallel with increasing early detection of breast cancer, DCIS rate increased up to 14–20%, and around half (68% in current study) of them would receive mastectomy. This group of patients constituted about 5% of annual breast cancer cases, which constituted a non-ignorable minority [6]. Current practice guideline had suggested SLNB to be performed in pre-OP biopsy diagnosed DCIS and received mastectomy as secondary lymph node staging surgery seems unreliable when post-OP pathology upgrade to invasive breast cancer [37, 38].

In our 430 patients with pre-OP biopsy diagnosed DCIS received mastectomy, 59.3% remained DCIS-post-OP, and in these patients SLNB seemed unnecessary or “over-treatment”. About 40.7%(175/430) of patients with pre-OP DCIS received mastectomy and upgrade to DCIS-IC post-OP. Among these 175 patients, 171 (98%) received SLNB, and 40 (23.5%) was found to have LN metastasis. This accounts for 9.8% (42/430) of pre-OP biopsy diagnosed DCIS and indicated mastectomy patients would have ALN metastasis. This result is consisted with Price et al.’s study, which showed about 10% patients would have ≧ 1 LN metastasis when SLNB was performed in patients with pre-OP biopsy diagnosed DCIS and indicated for mastectomy [6].

The reason that mastectomy patients tend to receive SLNB is that the upgrade rate is higher in patients received total mastectomy than partial mastectomy (BCS) (40.7% versus 23%, *P* < 0.01, Table 2). This is related to more multicentric breast cancer, larger tumor size of patients would receive mastectomy than BCS, and larger tumor or multicentric lesions would possess higher risk lead to upgrade to DCIS-IC. Among the predictors of ALN metastasis for patients received SLNB, patients with post-OP invasive component was the highest risk of lymph node metastasis (Table 5).

In current study, we tried to evaluate the pre-operative MRI for pre-OP DCIS patients, and we had 386 patients with detail ALN evaluation and post-operative pathologic report were analyzed for concordance. The accuracy of MRI: sensitivity 53.8%, specificity 77.8%, PPV 14.9%, NPV 95.9%, and accuracy 76.2%. This high NPV rate is very useful, especially for patients selected for BCS patients. In patients with MRI estimated tumor size ˂ 3 cm and lymph node-negative cases, the NPV of ALN metastasis is high (up to 96.4%), and all (3.6%, 5/137) the FN cases were limited to N1 (with 1–2 positive nodes only). Our data supported that pre-OP MRI evaluated node-negative patients suitable for BCS patients could safety omitted SLNB if whole breast radiotherapy is to be performed. In mastectomy patients, this high NPV could be used as shared decision making, however, could not guarantee metastatic free lymph node to omit SLNB (Table 2).

Our current study is limited in its retrospective study and limited cases, which could not answer whether subgroup of patients could omit axillary surgery in pre-OP DCIS patients indicated for mastectomy. Our current study, however, did collect of 682 pre-OP DCIS patients with detailed pre-op and post-OP lymph node pathologic report. Of special note that we had 386 patients with detailed pre-OP breast MRI evaluation and post-operative pathologic results, which enable us to evaluate the role of breast MRI in decision of lymph node surgery.

## Conclusion

Individualized lymph node evaluation/biopsy of pre-OP DCIS patients is warranted due to inherent specimen biopsy error, which leads to substantial upgrade to invasive component found in final pathology, and therefore raises the concern of axillary lymph node metastasis. Patients indicated for mastectomy deserved a more tailored planning as 50% SLNB was not necessary, 40% for staging purpose, and around 10% therapeutic. Pre-OP diagnosed DCIS patients with MRI tumor size < 3 cm and node-negative condition suitable for BCS could safely omit SLNB if whole breast radiotherapy is to be performed.

## Data Availability

The datasets generated and/or analyzed during the current study are not publicly available due to patients’ privacy but are available from the corresponding author on reasonable request.

## Abbreviations

ALN: Axillary lymph node
DCIS: Ductal carcinoma in situ
MRI: Magnetic resonance imaging
NPV: Negative predictive value
PPV: Positive predictive value
SLNB: Sentinel lymph node biopsy
ALND: Axillary lymph node dissection
CNB: Core needle biopsy
VACB: Vacuum-assisted core biopsy
IC: Invasive component
BCS: Breast conserving surgery

## References

[1] Heymans C, van Bastelaar J, Visschers RGJ, Vissers YLJ (2017). Sentinel node procedure obsolete in lumpectomy for ductal carcinoma in situ. Clin Breast Cancer.

[2] Caswell-Smith P, Wall M (2017). Ductal carcinoma in situ: is core needle biopsy ever enough?. J Med Imaging Radiat Oncol.

[3] Podoll MB, Reisenbichler ES, Roland L, Bruner A, Mizuguchi S, Sanders MAG (2018). Feasibility of the less is more approach in treating low-risk ductal carcinoma in situ diagnosed on core needle biopsy: ten-year review of ductal carcinoma in situ upgraded to invasion at surgery. Arch Pathol Lab Med.

[4] Munck F, Clausen EW, Balslev E, Kroman N, Tvedskov TF, Holm-Rasmussen EV (2020). Multicentre study of the risk of invasive cancer and use of sentinel node biopsy in women with a preoperative diagnosis of ductal carcinoma in situ. Br J Surg.

[5] Prendeville S, Ryan C, Feeley L, O’Connell F, Browne TJ, O’Sullivan MJ, Bennett MW. Sentinel lymph node biopsy is not warranted following a core needle biopsy diagnosis of ductal carcinoma in situ (DCIS) of the breast. Breast. 2015;24:197–200. 10.1016/j.breast.2015.01.004 25681861

[6] Price A, Schnabel F, Chun J, Kaplowitz E, Goodgal J, Guth A, Axelrod D, Shapiro R, Mema E, Moy L (2020). Sentinel lymph node positivity in patients undergoing mastectomies for ductal carcinoma in situ (DCIS). Breast J.

[7] Goyal A, Douglas-Jones A, Monypenny I, Sweetland H, Stevens G, Mansel RE (2006). Is there a role of sentinel lymph node biopsy in ductal carcinoma in situ?: analysis of 587 cases. Breast Cancer Res Treat.

[8] Yen TW, Hunt KK, Ross MI, Mirza NQ, Babiera GV, Meric-Bernstam F, Singletary SE, Symmans WF, Giordano SH, Feig BW (2005). Predictors of invasive breast cancer in patients with an initial diagnosis of ductal carcinoma in situ: a guide to selective use of sentinel lymph node biopsy in management of ductal carcinoma in situ. J Am Coll Surg.

[9] Shin YJ, Kim SM, Yun B, Jang M, Kim B, Lee SH (2019). Predictors of invasive breast cancer in patients with ductal carcinoma in situ in ultrasound-guided core needle biopsy. J Ultrasound Med.

[10] Nakhlis F, Harrison BT, Giess CS, Lester SC, Hughes KS, Coopey SB, King TA (2019). Evaluating the rate of upgrade to invasive breast cancer and/or ductal carcinoma in situ following a core biopsy diagnosis of non-classic lobular carcinoma in situ. Ann Surg Oncol.

[11] Lamb LR, Lehman CD, Oseni TO, Bahl M. Ductal carcinoma in situ (DCIS) at breast MRI: predictors of upgrade to invasive carcinoma. Acad Radiol. 2020;27:1994–9. 10.1016/j.acra.2019.09.025 31699638

[12] Lamb LR, Kim G, Oseni TO, Bahl M: Noncalcified ductal carcinoma in situ (DCIS): rate and predictors of upgrade to invasive carcinoma. Acad Radiol. 2021:28:e71–6. 10.1016/j.acra.2020.02.011 32222328

[13] Sim YT, Litherland J, Lindsay E, Hendry P, Brauer K, Dobson H, Cordiner C, Gagliardi T, Smart L (2015). Upgrade of ductal carcinoma in situ on core biopsies to invasive disease at final surgery: a retrospective review across the Scottish Breast Screening Programme. Clin Radiol.

[14] Chin-Lenn L, Mack LA, Temple W, Cherniak W, Quinn RR, Ravani P, Lewin AM, Quan ML (2014). Predictors of treatment with mastectomy, use of sentinel lymph node biopsy and upstaging to invasive cancer in patients diagnosed with breast ductal carcinoma in situ (DCIS) on core biopsy. Ann Surg Oncol.

[15] Sorrentino L, Sartani A, Bossi D, Amadori R, Nebuloni M, Truffi M, Bonzini M, Riggio E, Foschi D, Corsi F (2018). Sentinel node biopsy in ductal carcinoma in situ of the breast: Never justified?. Breast J.

[16] Watanabe Y, Anan K, Saimura M, Koga K, Fujino M, Mine M, Tamiya S, Nishihara K, Nakano T, Mitsuyama S (2018). Upstaging to invasive ductal carcinoma after mastectomy for ductal carcinoma in situ: predictive factors and role of sentinel lymph node biopsy. Breast Cancer.

[17] Allen A, Cauthen A, Dale P, Jean-Louis C, Lord A, Smith B (2019). Evaluating the frequency of upgrade to malignancy following surgical excision of high-risk breast lesions and ductal carcinoma in situ identified by core needle biopsy. Breast J.

[18] Verbelen H, Tjalma W, Meirte J, Gebruers N. Long-term morbidity after a negative sentinel node in breast cancer patients. Eur J Cancer Care (Engl). 2019;28:e13077. 10.1111/ecc.13077 31050088

[19] Verbelen H, Gebruers N, Eeckhout FM, Verlinden K, Tjalma W (2014). Shoulder and arm morbidity in sentinel node-negative breast cancer patients: a systematic review. Breast Cancer Res Treat.

[20] De Groef A, Van Kampen M, Tieto E, Schonweger P, Christiaens MR, Neven P, Geraerts I, Gebruers N, Devoogdt N (2016). Arm lymphoedema and upper limb impairments in sentinel node-negative breast cancer patients: a one year follow-up study. Breast.

[21] Edge SB, Sheldon DG. Counterpoint: sentinel lymph node biopsy is not indicated for ductal carcinoma in situ. J Natl Compr Canc Netw. 2003;1:207–12. 10.6004/jnccn.2003.0019 19768879

[22] Giuliano AE, Ballman KV, McCall L, Beitsch PD, Brennan MB, Kelemen PR, Ollila DW, Hansen NM, Whitworth PW, Blumencranz PW (2017). Effect of axillary dissection vs no axillary dissection on 10-year overall survival among women with invasive breast cancer and sentinel node metastasis: the ACOSOG Z0011 (Alliance) randomized clinical trial. JAMA.

[23] van Roozendaal LM, Goorts B, Klinkert M, Keymeulen K, De Vries B, Strobbe LJA, Wauters CAP, van Riet YE, Degreef E, Rutgers EJT (2016). Sentinel lymph node biopsy can be omitted in DCIS patients treated with breast conserving therapy. Breast Cancer Res Treat.

[24] Sakr R, Barranger E, Antoine M, Prugnolle H, Darai E, Uzan S (2006). Ductal carcinoma in situ: value of sentinel lymph node biopsy. J Surg Oncol.

[25] Bertozzi S, Cedolini C, Londero AP, Baita B, Giacomuzzi F, Capobianco D, Tortelli M, Uzzau A, Mariuzzi L, Risaliti A. Sentinel lymph node biopsy in patients affected by breast ductal carcinoma in situ with and without microinvasion: retrospective observational study. Medicine (Baltimore). 2019;98:e13831. 10.1097/MD.0000000000013831 PMC6344146 30608397

[26] Hotton J, Salleron J, Rauch P, Buhler J, Pierret M, Baumard F, Leufflen L, Marchal F. Predictive factors of axillary positive sentinel lymph node biopsy in extended ductal carcinoma in situ treated by simple mastectomy at once. J Gynecol Obstet Hum Reprod. 2020;49:101641. 10.1016/j.jogoh.2019.101641 31562936

[27] Magnoni F, Massari G, Santomauro G, Bagnardi V, Pagan E, Peruzzotti G, Galimberti V, Veronesi P, Sacchini VS (2019). Sentinel lymph node biopsy in microinvasive ductal carcinoma in situ. Br J Surg.

[28] Holm-Rasmussen EV, Jensen MB, Balslev E, Kroman N, Tvedskov TF (2018). Risk factors of sentinel and non-sentinel lymph node metastases in patients with ductal carcinoma in situ of the breast: A nationwide study. Breast.

[29] Carin AJ, Moliere S, Gabriele V, Lodi M, Thiebaut N, Neuberger K, Mathelin C (2017). Relevance of breast MRI in determining the size and focality of invasive breast cancer treated by mastectomy: a prospective study. World J Surg Oncol.

[30] Liu F, Wang M, Li H. Role of perfusion parameters on DCE-MRI and ADC values on DWMRI for invasive ductal carcinoma at 3.0 Tesla. World J Surg Oncol. 2018;16:239. 10.1186/s12957-018-1538-8 PMC6303963 30577820

[31] Wang LJ, Wu P, Li XX, Luo R, Wang DB, Guan WB (2018). Magnetic resonance imaging features for differentiating breast papilloma with high-risk or malignant lesions from benign papilloma: a retrospective study on 158 patients. World J Surg Oncol.

[32] Gao W, Guo N, Dong T (2018). Diffusion-weighted imaging in monitoring the pathological response to neoadjuvant chemotherapy in patients with breast cancer: a meta-analysis. World J Surg Oncol.

[33] Killelea BK, Grube BJ, Rishi M, Philpotts L, Tran EJ, Lannin DR (2013). Is the use of preoperative breast MRI predictive of mastectomy?. World J Surg Oncol.

[34] Lee CW, Wu HK, Lai HW, Wu WP, Chen ST, Chen DR, Chen CJ, Kuo SJ (2016). Preoperative clinicopathologic factors and breast magnetic resonance imaging features can predict ductal carcinoma in situ with invasive components. Eur J Radiol.

[35] Choi BB (2021). Dynamic contrast enhanced-MRI and diffusion-weighted image as predictors of lymphovascular invasion in node-negative invasive breast cancer. World J Surg Oncol.

[36] Lyman GH, Somerfield MR, Bosserman LD, Perkins CL, Weaver DL, Giuliano AE (2017). Sentinel lymph node biopsy for patients with early-stage breast cancer: American Society of Clinical Oncology Clinical Practice Guideline Update. J Clin Oncol.

[37] Cody HS, Zee KJV: Point: Sentinel lymph node biopsy is indicated for patients with DCIS. J Natl Compr Canc New. 2003;1:199–206. 10.6004/jnccn.2003.0018 19768878

[38] Cardoso F, Kyriakides S, Ohno S, Penault-Llorca F, Poortmans P, Rubio IT, Zackrisson S, Senkus E, clinicalguidelines@esmo.org EGCEa: Early breast cancer: ESMO Clinical Practice Guidelines for diagnosis, treatment and follow-updagger. Ann Oncol. 2019;30:1194–220. 10.1093/annonc/mdz173 31161190

